# A Comparative Study of Regression Methods for Solving the Timepix Calibration Task

**DOI:** 10.3390/s25216714

**Published:** 2025-11-03

**Authors:** Jan Broulím, Matěj Prokop, Libor Nouzák, Pavel Smrčka

**Affiliations:** 1 Czech Technical University, Jugoslavskych Partyzanu 1580/3, 16000 Prague, Czech Republic; prokom12@fjfi.cvut.cz (M.P.); smrcka@fbmi.cvut.cz (P.S.); 2 Charles University, Ovocny trh 560/5, 11636 Prague, Czech Republic; libor.nouzak@mff.cuni.cz

**Keywords:** Timepix, Timepix3, Timepix4, Levenberg–Marquardt, regression, optimization algorithms, variable projection, calibration

## Abstract

In this article, we provide a study of the energy calibration model used for Timepix-type detectors. The Timepix detectors, operating in Time-over-Threshold mode, measure information that needs to be mapped into the corresponding energies using a non-linear function. We consider three iterative algorithms, Gradient-Descent, Gauss–Newton and Levenberg–Marquardt algorithm, which we modify according to the calibration model constraints to perform better in terms of the convergence properties. Moreover, based on the variable projection method, we suggest a partial linearization of the calibration problem and provide results for this novel method.

## 1. Introduction

The goal was to create an efficient and robust algorithm for fitting the energy Time-over-Threshold (ToT) calibration curve to data measured by a specific pixel of the Timepix-type detector. The problem requires constrained non-linear regression, for which several algorithms were implemented.

This article provides calibration solutions based on various mathematical algorithms and validates the effectiveness of the calibration.

### 1.1. Timepix Detectors

Timepix-type detectors [1,2,3,4] are hybrid pixel detectors based on the readout chip [1] and a bump-bonded sensor layer, typically made of Si, SiC, GaAs, or CdTe. They contain a matrix of pixels with the size of 55 μm × 55 μm per pixel and a granularity of 256 × 256 for Timepix, Timepix2, and Timepix3, or 512 × 448 for Timepix4. Each pixel represents an independent spectroscopic channel, providing information about the energy and time of acquired events in terms of the ToT and the Time of Arrival (ToA) values. Since the Timepix3 generation, both values can be measured simultaneously [3,5,6].

**Figure 1:**
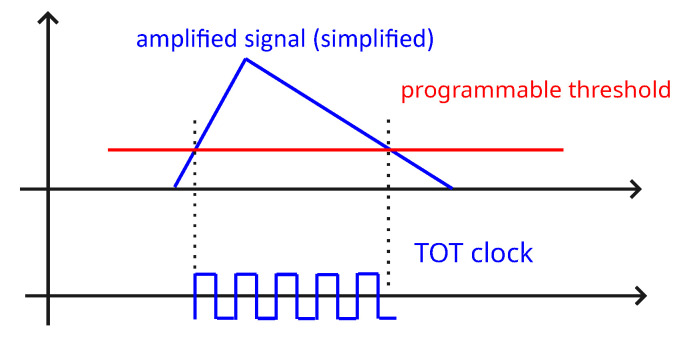
A simplified principle of measuring in the ToT mode.

**Figure 2:**
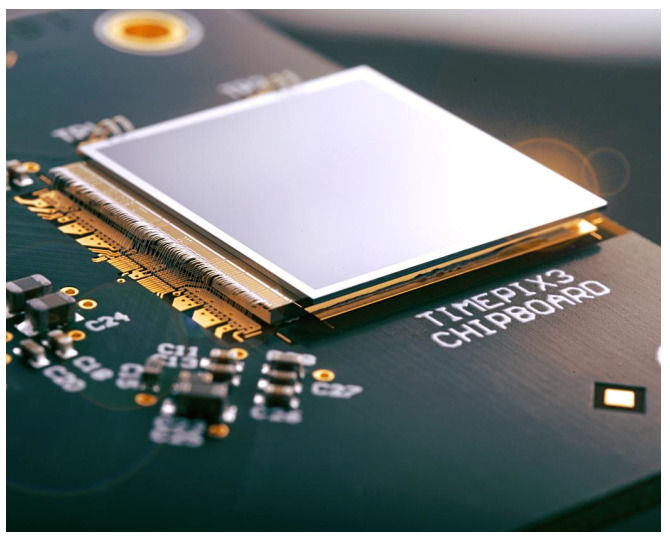
The Timepix3 wire-bonded on the chipboard [7].

When measuring in the ToT mode, the detector counts the number of clock cycles during a certain period depending on the programmable threshold and deposited energy. The ToT value represents the incremental number of clock cycles during which the incoming signal remains above a settable threshold. The signal consists of two parts: a rapid rise as a result of charging the capacitor, followed by a slower discharge. The amplitude is proportional to the energy deposited. When the amplified signal exceeds the settable threshold, the ToT counter is incremented based on the ticks of the clock. When the signal is below the threshold, it does not contribute to the measurement, which introduces non-linearity in the calibration function. This process is illustrated in Figure 1. The wire-bonded sensor is shown in Figure 2.

The calibration setup, measurement conditions, task definition, and algorithms used are listed below.

### 1.2. Calibration Setup

The setup consists of an X-ray tube, metal foils, and a Timepix detector. The X-ray tube irradiates the metal foil, producing characteristic X-rays at known energies. The characteristic X-rays are acquired by the Timepix detector. Alternatively, the small calibration sources are used.

Pixel detectors exploit the charge-sharing effect [8], which means that the deposited energy is spread over multiple pixels. This creates multi-pixel events, which we call clusters. Based on their morphology, we can distinguish the type of particle [9,10,11]. The sum of the energies per cluster (track) corresponds to the total deposited energy. This type of calibration is applied to the energies up to 1 MeV, before the volcano effect appears [12,13,14]. The volcano effect is caused by saturation within the pixel ASIC, resulting in two pulses in the preamplifier. The calibration fit for these energies differs from the equation used in this paper. The term volcano refers to the blobs observed from heavy, high-energy particles, which show lower acquired values at their centers.

In this work, we are interested in single-pixel events, where all deposited energy is acquired within a single pixel. This is common for X-rays. The measured value, called Time-over-Threshold (ToT), is mapped onto a known energy expressed in keV. A filter to exclude multi-pixel events is applied. The filter searches for hits in neighboring pixels within a given time window, and those hits are deleted.

We acquired several datasets; each dataset corresponds to a material producing X-rays (Cd, Ti, Cu, and Zr) and another dataset for the Am-241 source. Since we are interested only in single-pixel events, we can consider that measurements were taken at 65,536 channels, which corresponds to the matrix size. For five calibration points, this results in five datasets, each measured at 65,536 channels. The Fe-55 dataset was dropped due to low statistics. We also worked with the second detector to confirm and validate the measurements.

**Figure 3:**
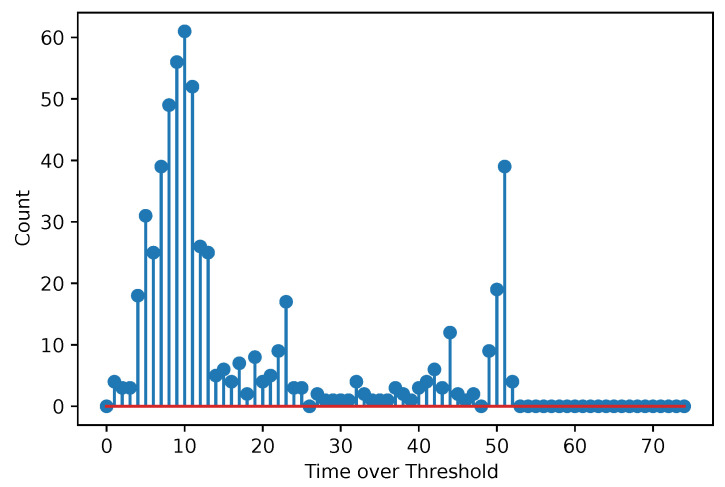
Acquired points based on the measurement with the Am-241 source. The spectrum shows the ToT values for one selected pixel. The red color represents the baseline (also in the following figures).

**Figure 4:**
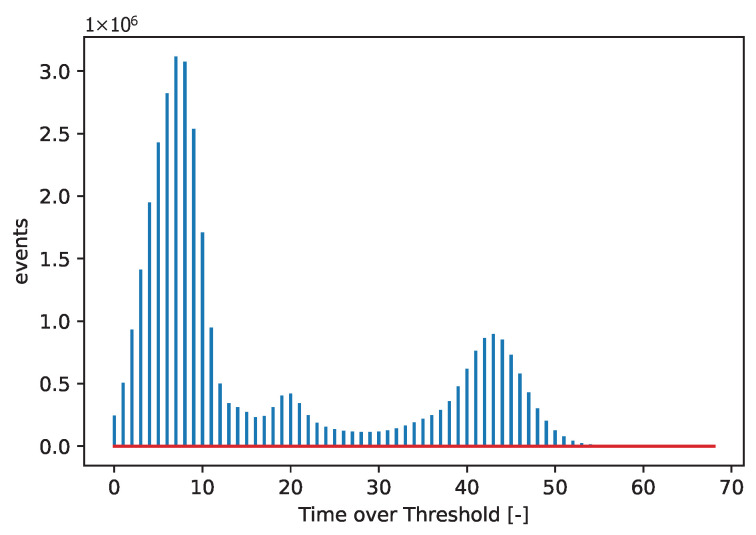
Acquired points based on the measurement with the Am-241 source. The spectrum shows a summation of ToT values for all pixels.

**Figure 5:**
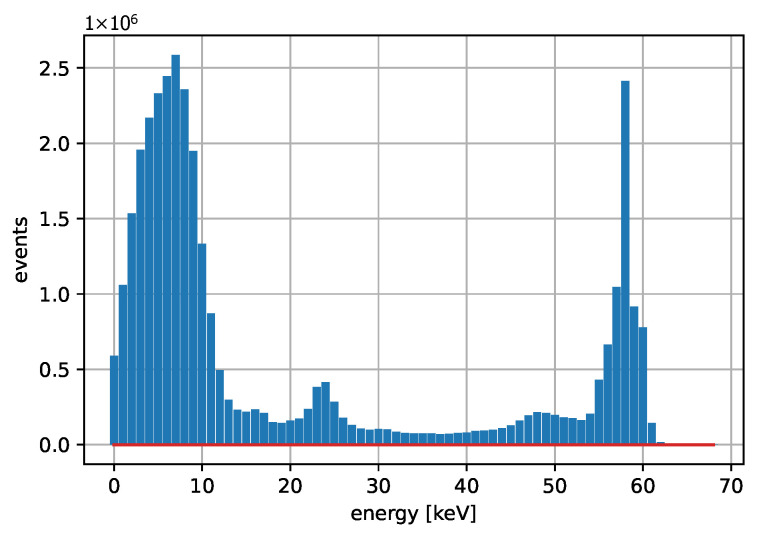
Acquired data points are based on measurements with the Am-241 source after the calibration. The spectrum represents the sum of the energy values from all pixels, expressed in keV. A clear 59.6 keV peak is observed, which appears significantly narrower when compared to the corresponding ToT values.

**Figure 6:**
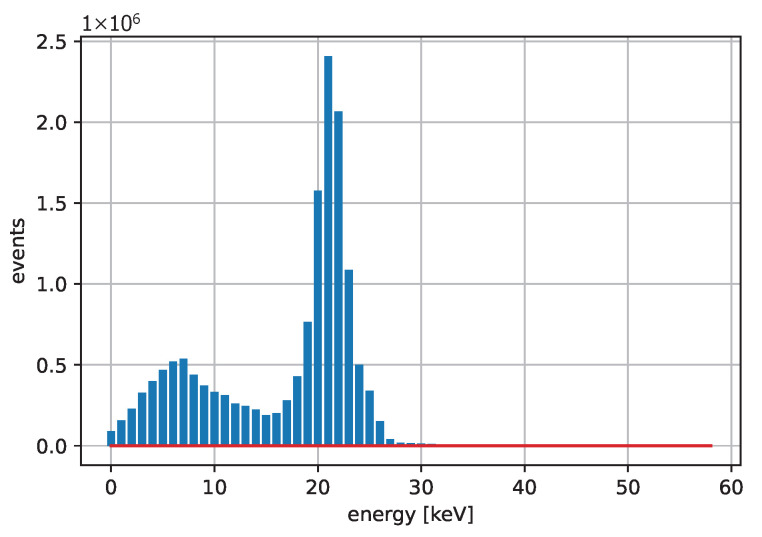
The spectrum obtained for Cd producing characteristic X-rays. All pixels are summed up.

Figure 3 and Figure 4 show acquired histograms of ToT values for Am-241. We are interested in the last peak, which corresponds to the full energy deposition at 59.6 keV, which is around the maximum energy for single pixel hits in the Si sensor used. Other peaks and Compton scattering can also be identified in the spectrum. Figure 3 depicts values acquired within one selected pixel, while Figure 4 shows the sum of events in the entire matrix. The spectra after the calibration, i.e., when ToT is recalculated to energies, are plotted in Figure 5 for Am-241 and in Figure 6 for Cd irradiated by the X-ray tube. The principle of the calibration is described in the following section, and the energies for these spectra are obtained using Equation (29). The energy resolution of the detector is expressed using σ or Full Width at Half Maximum (FWHM), which quantifies the peak widths at particular energies.

Table 1 and Table 2 list the calibration energies. For calibration purposes, we are interested in the peak positions. We can search for the maximal values or fit peaks with the use of a Gaussian approximation, which may provide better results by means of estimating peak positions between the two ToT integer values. This approach can produce slightly better results compared to the method that searches for the maximal values within histograms.

## 2. Task Definition

The task is to model the dependence between measured ToT values and the energy expressed in keV. According to the detector description and the electronics used we expect the following relation [15,16,17,18,19,20]:

(1) $$y=f\left(x,\beta \right)=\beta_{a}x+\beta_{b}-\frac{\beta_{c}}{x-\beta_{d}}$$

where $x=(x_{1},x_{2},\ldots ,x_{n})$ are the measured energies expressed in keV, $y=(y_{1},y_{2},\ldots ,y_{n})$ are the corresponding measured ToT values (non-negative). This equation is not valid at high energies (above 1 MeV), where ASIC saturation occurs. The calibration fit in this region is almost linear, but higher energies correspond to lower ToT values.

The task is to determine coefficients $\beta =(\beta_{a},\beta_{b},\beta_{c},\beta_{d})$ that minimize the value of the cost function F(β), given by the RSS value (Residual Sum of Squares):

(2) $$F\left(\beta \right)=\sum_{i=1}^{n}r_{i}^{2}=\sum_{i=1}^{n}{\left(y_{i}-f\left(x_{i},\beta \right)\right)}^{2}.$$

The values $r_{i}=y_{i}-f\left(x_{i},\beta \right)$ are referred to as *i*-th component of the residual vector r.

The value of $\beta_{d}$ solution is limited by the condition that it should be less than the minimum of a measured calibration point, as follows:

(3) $$\beta_{d}<\min_{i}\left(x_{i}\right)$$

Also, large negative values can signal a problematic solution. The value of $\beta_{a}$ and $\beta_{c}$ are expected to be positive, written as follows:

(4) $$\beta_{a}>0,$$

(5) $$\beta_{c}>0.$$

## 3. Iterative Algorithms

We have evaluated the performance of the following three algorithms [21,22] for calibrating detectors:

- Gradient Descent (GD),
- Levenberg–Marquardt (LM),
- Gauss–Newton (GN).

Iterative algorithms start with setting the initial guess $\beta^{\left(0\right)}$. Then, they iterate in order to update the current estimate, with the goal to lower the cost function. The next iteration estimate during the algorithm run is calculated as follows:

(6) $$\beta^{k+1}=\beta^{k}+\delta^{k},$$

where $\delta^{k}$ is the step updating *k*-th iteration estimate. Iterative algorithms differ in the way of calculating $\delta^{k}$.

### 3.1. Gradient Descent

A gradient descent is the algorithm for finding the local maximum or minimum of a given function. We minimize the Function (2), based on performing steps against its gradient. The value of $\delta^{\left(k\right)}$ for the gradient descent algorithm is given by the equation:

(7) $$\delta^{\left(k\right)}=-\gamma^{\left(k\right)}\nabla F{\left(\beta \right)}^{\left(k\right)},$$

where γ is called the step size. In the simplest case, it could have a fixed value, independent of *k*. However, for better performance, we use a backtracking line search to find the value of the step size [23]. For simplification, we omit *k*, denoting the current iteration index, in the following expressions. At the beginning of each iteration, we start with $\gamma_{0}$ (the maximum step size), which is a fixed algorithm parameter. Then we multiply γ with the fixed adjusting constant τ<1, written as follows:

(8) $$\gamma_{i+1}=\tau \gamma_{i},$$

while the condition given by (Armijo–Goldstein condition negation) [23]

(9) $$F\left(\beta -\gamma \nabla F\left(\beta \right)\right)>F\left(\beta \right)-\frac{1}{2}{\gamma ||\nabla F\left(\beta \right)||}^{2},$$

is satisfied. When this process is finished, we have found our best step size value estimate, and we perform the step with this value. This method provides a general approach for solving the tasks; however, other methods exhibit better convergence properties, as shown in the following figures.

### 3.2. Gauss–Newton

The Gauss–Newton algorithm is used for solving non-linear least squares problems. Given the residual functions ***r*** = $\left(r_{1},r_{2},\ldots ,r_{m}\right)$ and variables $\beta =(\beta_{i}$), the value of $\delta^{\left(k\right)}$ is found by solving the matrix equation given by the following:

(10) $$\left(J^{T}J\right)\delta^{\left(k\right)}=J^{T}r^{\left(k\right)}.$$

Assuming Equation (6), we can express the next β values as follows:

(11) $$\beta^{k+1}=\beta^{k}+{\left(J^{T}J\right)}^{-1}J^{T}r^{\left(k\right)}.$$

where J is the Jacobian matrix. The Formula (10) allows us to find desired values directly from solving the system of linear equations, which can be performed faster than the calculation of the inverse matrix (11).

### 3.3. Levenberg–Marquardt (LM)

The idea of Levenberg–Marquardt is to improve the convergence of the Gauss–Newton method by introducing a damping parameter [22]. The algorithm is based on the δ calculation from the matrix equation given by the following:

(12) $$\left(J^{T}J+\lambda^{\left(k\right)}I\right)\delta^{\left(k\right)}=J^{T}r^{\left(k\right)},$$

where I is the identity matrix and λ is called the damping parameter.

For the adaptation of the damping parameter, we use the method proposed by Marquardt [22]. Let υ>1 be the adjusting constant of the algorithm, and let $\lambda^{0}$ be the initial damping parameter of the algorithm.

At the beginning of the *k*-th iteration, let $\lambda^{\left(k-1\right)}$ be the damping parameter value from the previous iteration, or we set $\lambda^{\left(-1\right)}=\lambda_{initial}$ for the first iteration. Our goal is to find the best damping parameter for the current iteration, denoted as $\lambda^{\left(k\right)}$ (k=0,1,2,…). Consider the cost function $F_{\lambda^{\left(k-1\right)}/\upsilon }$ calculated for $\lambda^{\left(k-1\right)}/\upsilon$. Then the following algorithm is used [22]:

- If $F_{\lambda^{\left(k-1\right)}/\upsilon }\leq F_{\lambda^{\left(k-1\right)}}$ we set $\lambda^{\left(k\right)}=\lambda^{\left(k-1\right)}/\upsilon$;
- If $F_{\lambda^{\left(k-1\right)}/\upsilon }>F_{\lambda^{\left(k-1\right)}}$ and $F_{\lambda^{\left(k-1\right)}}\leq F\left(\beta^{\left(k\right)}\right)$ we set $\lambda^{\left(k\right)}=\lambda^{\left(k-1\right)}$;
- If $F_{\lambda^{\left(k-1\right)}/\upsilon }>F_{\lambda^{\left(k-1\right)}}$ and $F_{\lambda^{\left(k-1\right)}}>F\left(\beta^{\left(k\right)}\right)$ we multiply $\lambda^{\left(k-1\right)}$ by υ for some smallest *w* until $F_{\lambda^{\left(k-1\right)}\upsilon^{w}}\leq F\left(\beta^{\left(k\right)}\right)$ is satisfied. Then we set $\lambda^{\left(k\right)}=\lambda^{\left(k-1\right)}\upsilon^{w}$.

### 3.4. Analytical Derivations Used for Calculations

Here we derive partial derivatives, which are required for the algorithm implementations.

#### 3.4.1. Jacobian Matrix

Jacobian matrix J is a matrix whose columns are the gradients of the regression function for the *i*-th data point. That is described by the following equations:

(13) $$J_{i1}=\frac{\partial f}{\partial \beta_{a}}\left(x_{i},\beta \right)$$

(14) $$J_{i2}=\frac{\partial f}{\partial \beta_{b}}\left(x_{i},\beta \right)$$

(15) $$J_{i3}=\frac{\partial f}{\partial \beta_{c}}\left(x_{i},\beta \right)$$

(16) $$J_{i4}=\frac{\partial f}{\partial \beta_{d}}\left(x_{i},\beta \right),$$

where the partial derivatives within the Jacobian matrix are expressed as

(17) $$\frac{\partial f}{\partial \beta_{a}}\left(x_{i},\beta \right)=x_{i}$$

(18) $$\frac{\partial f}{\partial \beta_{b}}\left(x_{i},\beta \right)=1$$

(19) $$\frac{\partial f}{\partial \beta_{c}}\left(x_{i},\beta \right)=-\frac{1}{x_{i}-\beta_{d}}$$

(20) $$\frac{\partial f}{\partial \beta_{d}}\left(x_{i},\beta \right)=-\frac{\beta_{c}}{{\left(x_{i}-\beta_{d}\right)}^{2}}$$

#### 3.4.2. Gradient of the Cost Function

The analytic expression for components of the cost function gradient can be found by the differentiation of Equation (2).

(21) $$\begin{array}{c} \frac{\partial F}{\partial \beta_{a}}\left(\beta \right)=-2\sum_{i=1}^{n}x_{i}y_{i}+2\beta_{a}\sum_{i=1}^{n}x_{i}^{2}+ \end{array}$$

(22) $$\begin{array}{c} +2\beta_{b}\sum_{i=1}^{n}x_{i}-2\beta_{c}\sum_{i=1}^{n}\frac{x_{i}}{x_{i}-\beta_{d}} \end{array}$$

(23) $$\begin{array}{c} \frac{\partial F}{\partial \beta_{b}}\left(\beta \right)=-2\sum_{i=1}^{n}y_{i}+2\beta_{a}\sum_{i=1}^{n}x_{i}+ \end{array}$$

(24) $$\begin{array}{c} +2\beta_{b}n-2\beta_{c}\sum_{i=1}^{n}\frac{1}{x_{i}-\beta_{d}} \end{array}$$

(25) $$\begin{array}{c} \frac{\partial F}{\partial \beta_{c}}\left(\beta \right)=2\sum_{i=1}^{n}\frac{y_{i}}{x_{i}-\beta_{d}}-2\beta_{a}\sum_{i=1}^{n}\frac{x_{i}}{x_{i}-\beta_{d}}- \end{array}$$

(26) $$\begin{array}{c} -2\beta_{b}\sum_{i=1}^{n}\frac{1}{x_{i}-\beta_{d}}+2\beta_{c}\sum_{i=1}^{n}\frac{1}{{\left(x_{i}-\beta_{d}\right)}^{2}} \end{array}$$

(27) $$\begin{array}{c} \frac{\partial F}{\partial \beta_{d}}\left(\beta \right)=2\beta_{c}\sum_{i=1}^{n}\frac{y_{i}}{{\left(x_{i}-\beta_{d}\right)}^{2}}-2\beta_{a}\beta_{c}\sum_{i=1}^{n}\frac{x_{i}}{{\left(x_{i}-\beta_{d}\right)}^{2}} \end{array}$$

(28) $$\begin{array}{c} -2\beta_{b}\beta_{c}\sum_{i=1}^{n}\frac{1}{{\left(x_{i}-\beta_{d}\right)}^{2}}+2\beta_{c}^{2}\sum_{i=1}^{n}\frac{1}{{\left(x_{i}-\beta_{d}\right)}^{3}} \end{array}$$

The sums from this equation that are independent of β can be precomputed. The sums dependent on β must be computed again with every iteration.

### 3.5. Algorithm Modifications

We modify the algorithms to have better convergence properties. We must consider that the algorithm can converge to a different minimum than the desired one.

We must avoid cases where the algorithm terminates outside the expected bounds by properly identifying poor convergence behavior through consideration of the bounds (3)–(5). For improving convergence and enlarging the region of initial estimates, which terminates in good solutions, we suggest using *randomization* step during the algorithm run. If the β vector goes out of the given bounds, random numbers are added to its elements.

All iterative algorithms are performed within a cycle until the maximal number of iterations is reached. This stopping condition is further extended by checking the convergence criteria, e.g., the gradient of the cost function or the iteration step size. A Gauss–Newton algorithm is described in terms of pseudo code in Algorithm 1. We calculate δ according to Equation (10) in order to avoid the calculation of the inverse matrix when β is expressed directly Equation (11). The values of the J matrix are expressed analytically. The Gradient-Descent algorithm implementation is an intuitive modification of the pseudo code. Algorithm 2 shows the LM regression, extended by our randomization. Compared to the GN algorithm, it uses a damping parameter to improve the convergence. Both algorithms provide β coefficients as the output.

The comparative results for the algorithms are shown in the following sections. The cases of wrong convergence, incorrect $\beta_{d}$ values, and the algorithm comparisons are also included in the study.
**Algorithm 1** Iterative algorithm—GN.  1:**input:**$\;\beta_{0}$  2:**output:** β                                   {vector of 4 coefficients}
  3:$\beta \leftarrow \beta_{0}$;                        {initial guesses, initial damping parameter}  4:k←0;                                            {iteration index}  5:**while** ($k<k_{max}$) **do**  6:    $\delta \leftarrow \mathrm{solve}\;J^{T}J\delta =J^{T}r$  7:    β←β+δ  8:    **if** stoppingCriterion(δ,β) **then**  9:       **break** {stops updating if the delta value or the gradient of the cost function is below a given threshold}10:    **end if**11:    k←k+112:**end while**13:**return** β

**Algorithm 2** Iterative algorithm—LM.
  1:**input:** $\lambda_{initial}$; ν; $\beta_{0}$  2:**output:** β                                   {vector of 4 coefficients}  3:$\beta \leftarrow \beta_{0}$; $\lambda \leftarrow \lambda_{initial}$;                      {initial guesses, initial damping parameter}  4:k←0;                                             {iteration index}  5:**while** ($k<k_{max}$) **do**  6:    $J^{T}\leftarrow \mathrm{jacobianTransposed}\left(\beta \right)$  7:    r←residualsVector(β)  8:    $\delta_{1}\leftarrow \mathrm{solve}\;\left(J^{T}J+\lambda I\right)\delta =J^{T}r$  9:    $\delta_{2}\leftarrow \mathrm{solve}\;\left(J^{T}J+\frac{\lambda }{\upsilon }I\right)\delta =J^{T}r$10:    **if** $F\left(\beta +\delta_{1}\right)\geq F\left(\beta +\delta_{2}\right)$ **then**11:        $\lambda \leftarrow \frac{\lambda }{\upsilon }$12:        $\beta \leftarrow \beta +\delta_{2}$                         {a part specific for LM regression}13:    **else if** $F\left(\beta +\delta_{1}\right)\leq F\left(\beta \right)$ **then**14:        $\beta \leftarrow \beta +\delta_{1}$15:    **else**16:        **while** $F\left(\beta +\delta_{1}\right)>F\left(\beta \right)$ **do**17:            λ←λυ18:            $\delta_{1}\leftarrow \mathrm{solve}\;\left(J^{T}J+\lambda I\right)\delta =J^{T}r$ {this is recommended to limit by the maximum number of steps}19:        **end while**20:        $\beta \leftarrow \beta +\delta_{1}$21:    **end if**22:    **if** !insideConstraints(β) **then**23:        β←randomize()24:    **else if** isSolution(β) **then**25:        **return** β26:    **end if**27:    k←k+128:
**end while**
29:
**return**



## 4. Results for Iterative Algorithms

**Figure 7:**
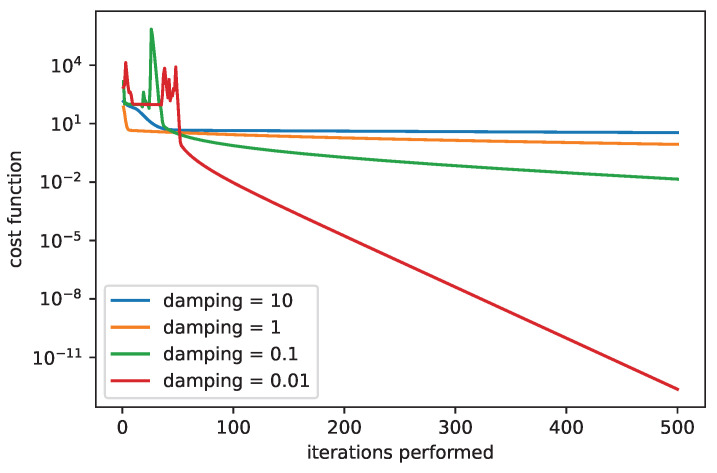
The convergence of LM algorithm, comparison for various damping parameters, $\beta_{0}=\left(0,0,25,1\right)$.

Figure 7 shows a comparison for the LM algorithm when sweeping with the damping parameter. We can see that the high values of the damping parameter can slow down the convergence. While the damping parameter equal to 10 provides very slow but stable convergence, the damping parameter equal to 0.01 results in unstable convergence at the beginning, followed by a rapid decrease in the cost function. The introduction of the damping parameter extends the GN algorithm and improves its properties.

**Figure 8:**
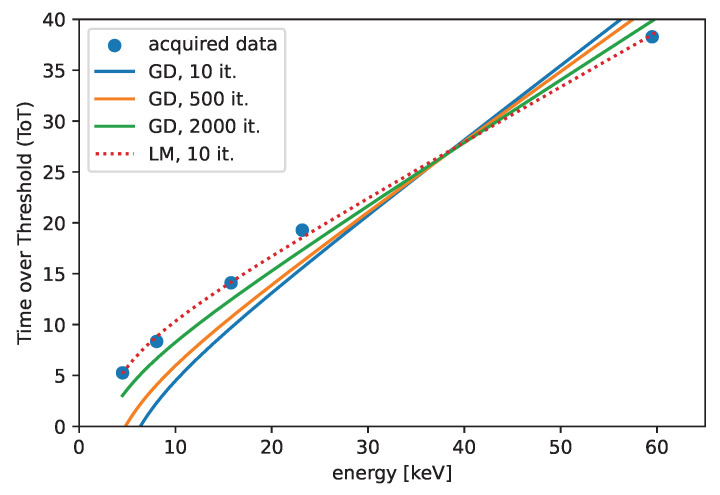
The convergence of the GD algorithm, comparison with the LM algorithm. The initial guesses are set to $\beta_{0}=\left(0,0,25,1\right)$.

The fitted curve is shown in Figure 8 for the two different algorithms. The comparison depicts the iteration count dependence of the fitted curve for the GD and the LM algorithms. We can recognize that the convergence of the LM algorithm is much faster than the convergence of the GD algorithm. A significant difference is observed in the comparison, where the LM algorithm performed better after 50 iterations compared to the GD algorithm after 2000 iterations.

**Figure 9:**
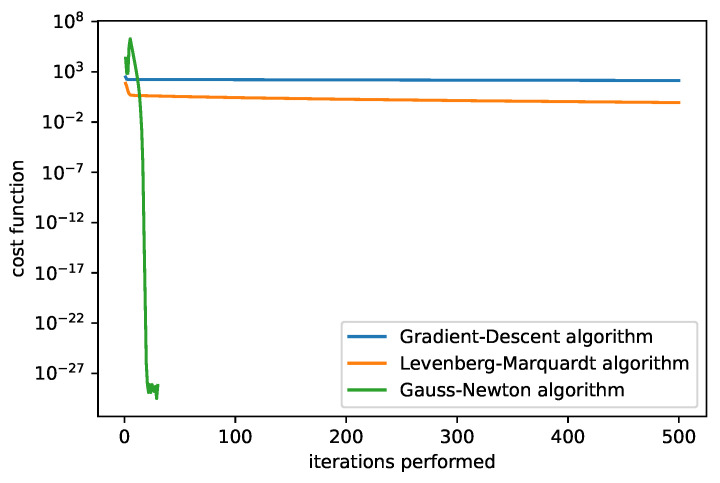
The cost function of algorithms. The initial guesses set to $\beta_{0}=\left(0,0,25,1\right)$, damping parameter = 1, rate = 0.0001. We can see that the Gauss–Newton algorithm can in some cases converge very quickly.

**Figure 10:**
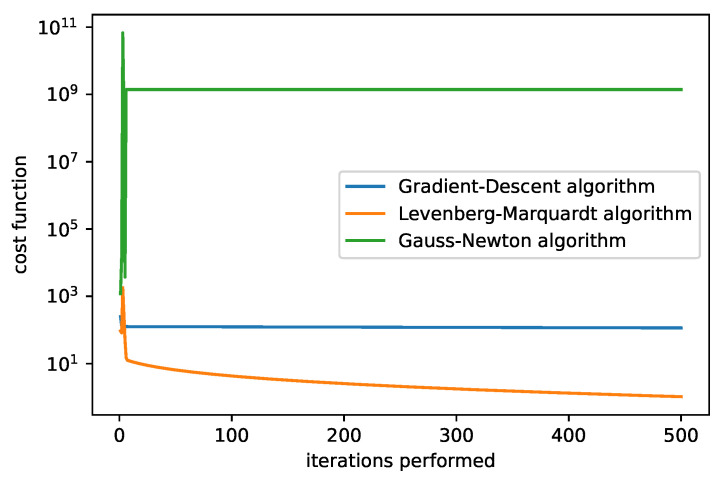
The cost function of the algorithms. The initial guesses are set to $\beta_{0}=\left(0,0,5,1\right)$, damping parameter = 1, and rate = 0.0001. We can recognize the best performance of the LM algorithm.

**Figure 11:**
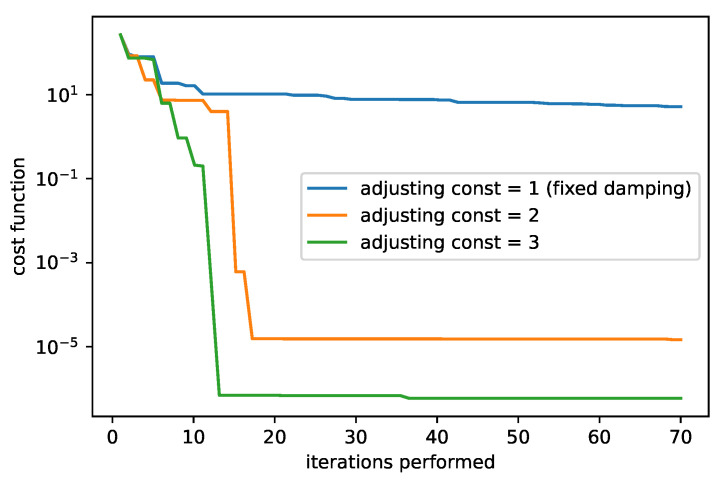
The cost function of the LM algorithm with the different adjusting constants. The initial guesses set to $\beta_{0}=\left(1,1,5,1\right)$ are used and the initial damping is set to 1.

In Figure 9 and Figure 10, we compare the dependency of the cost function value on the iterations performed. We show results for two different initial guesses. Figure 9 corresponds to the initial guess $\beta_{0}=\left(0,0,25,1\right)$, Figure 10 to $\beta_{0}=\left(0,0,5,1\right)$. In the first case the LM algorithm produced the best results (with the fixed damping used). A comparison of the different adjusting constants for the LM algorithm is plotted in Figure 11 with the initial guess $\beta_{0}=\left(1,1,5,1\right)$. The filter that selects the minimum cost up to the current iteration is applied.

**Figure 12:**
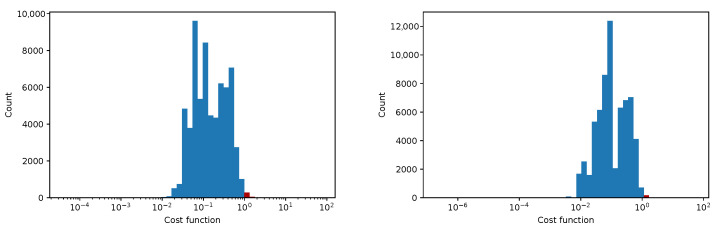
The convergence of LM algorithm after 50 iterations, all pixels included, a comparison for various damping parameters is shown, the initial guesses were $\beta_{0}=\left(0,0,0,1\right)$. The (**left**) plot shows fixed steps, the (**right**) picture uses adjusting constant equal to 5.

**Figure 13:**
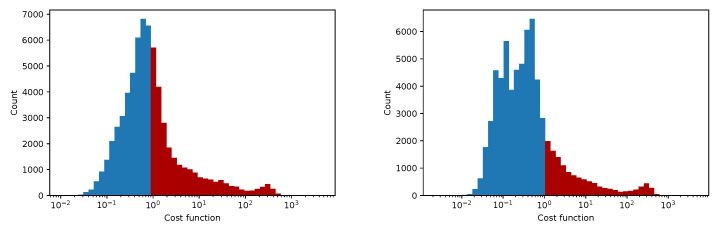
The convergence of the LM algorithm after 5 iterations, all pixels are included, a comparison for various damping parameters is shown, the initial guesses were $\beta_{0}=\left(1,1,25,1\right)$. The (**left**) plot shows the fixed steps, the (**right**) picture uses adjusting constant equal to 3.

**Figure 14:**
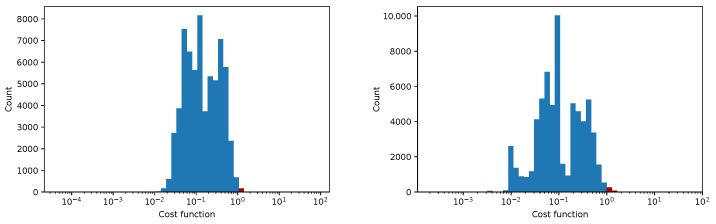
The convergence of the LM algorithm after 50 iterations, all pixels are included, a comparison for various damping parameters is shown, the initial guesses were $\beta_{0}=\left(1,1,25,1\right)$. The (**left**) plot shows fixed steps, the (**right**) picture uses adjusting constant equal to 3.

**Figure 15:**
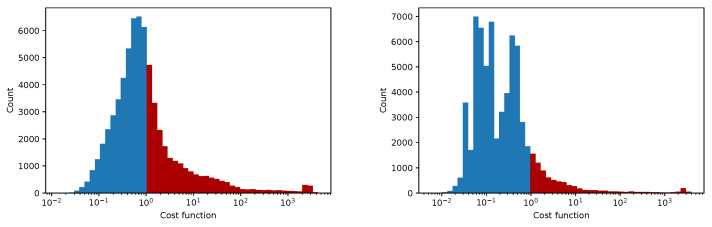
The convergence of the LM algorithm after 5 iterations, all pixels are included, comparison for various damping parameters is shown, the initial guesses were $\beta_{0}=\left(0,0,0,1\right)$. The (**left**) plot shows fixed steps, the (**right**) picture uses adjusting constant equal to 5.

**Figure 16:**
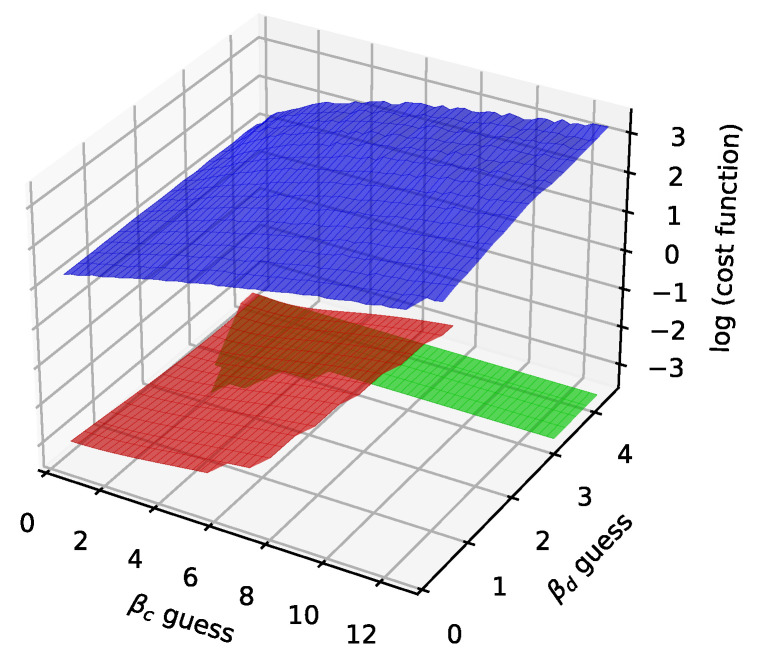
The convergence plot for the three different algorithms after 100 iterations. The red color stands for the LM algorithm (fixed damping 1), the green color for the GN algorithm, the blue color for the GD algorithm (maximal rate 100, adjusting 0.5). The initial guesses for $\beta_{a}=1$, $\beta_{b}=1$. Figures 29–31.

**Figure 17:**
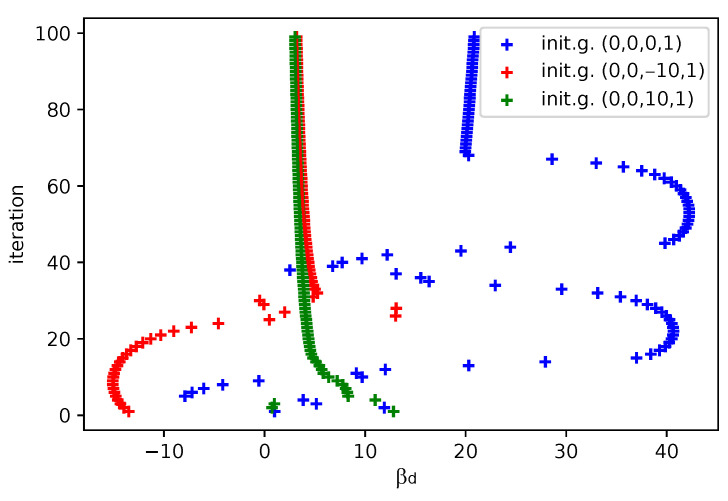
Visualization of the convergence trajectory in terms of $\beta_{d}$ value, the LM algorithm is used. The blue trajectory shows a failure in convergence, however, it can be easily identified as $\beta_{d}$ cannot be between measured points.

In Figure 12, Figure 13, Figure 14 and Figure 15 we show the histogram of the cost function for all pixels. Figure 13 and Figure 15 show results after 5 iterations performed. The convergence behavior of the three algorithms was illustrated in Figure 16 in terms of the 3D plot. The convergence trajectory is indicated in Figure 17.

### Energy Spectra

The calibration function converting the energy *E* to the ToT value is expressed in Equation (1).

We denote t=F(E), $E>\beta_{d}$, and derive the inversion function as follows:

(29) $$E=\frac{t+\beta_{a}\beta_{d}-\beta_{b}\pm \sqrt{{\left(t-\beta_{a}\beta_{d}-\beta_{b}\right)}^{2}+4\beta_{a}\beta_{c}}}{2\beta_{a}},$$

where *t* is the ToT value.

Roots of the quadratic formula corresponds to positive and negative brach of the calibration function, $E_{+}\geq \beta_{d}$ and $E_{-}\leq \beta_{d}$.

**Figure 18:**
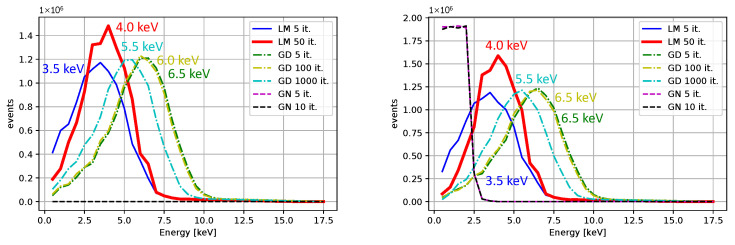
The measurement with Ti after the calibration. The initial guesses were $\beta_{0}=\left(0,0,0,0\right)$ for the left plot and $\beta_{0}=\left(5,0,0,1\right)$. The peak maxima with respect to the binning are indicated (no fitting was applied).

**Figure 19:**
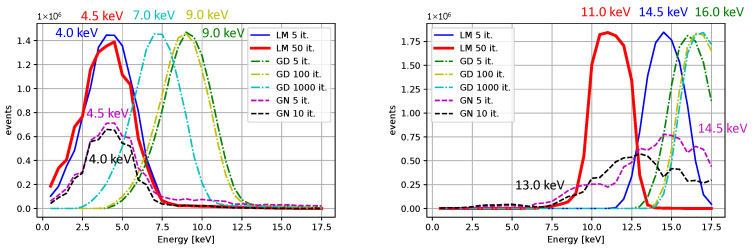
The measurement with Ti after the calibration. The initial guesses were $\beta_{0}=\left(0,0,25,0\right)$ for the left plot and $\beta_{0}=\left(0,0,50,10\right)$. The peak maxima with respect to the binning are indicated (no fitting was applied).

**Figure 20:**
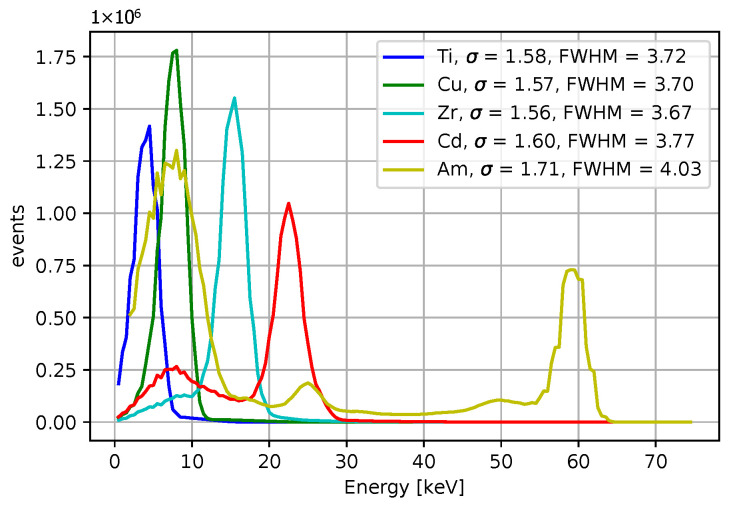
The energy spectrum for 4 X-rays datasets measured X-rays and the Am-241 source. The LM algorithm at 50 iterations was used, the initial guess were $\beta_{0}=\left(0,0,20,0\right)$.

**Figure 21:**
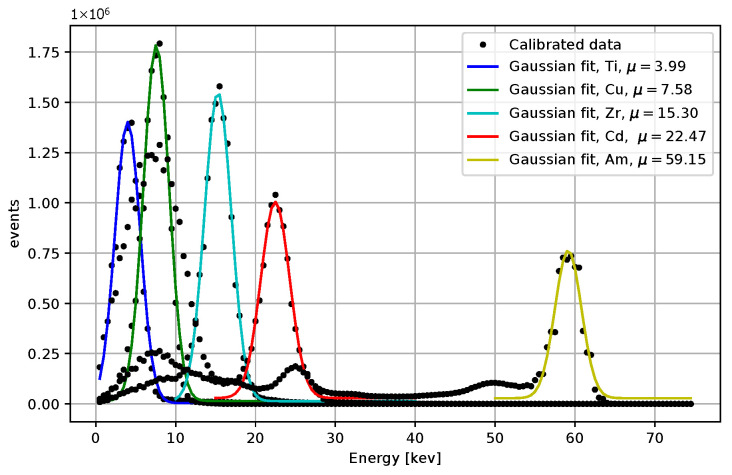
Gaussian fits for the energy spectrum for 4 X-rays datasets measured X-rays and the Am-241 source. The LM algorithm at 50 iterations was used, the initial guess were $\beta_{0}=\left(0,0,20,0\right)$. The peak centers obtained from the fits are included.

Comparisons for different iterative algorithms and different numbers of iterations are shown in Figure 18 and Figure 19, with Ti as the source of X-rays. The center value of the peak for Ti, determined using the LM algorithm with 50 iterations, is located between 4.0 and 4.5 keV for all initial guesses except for $\beta_{0}=\left(0,0,50,10\right)$. Considering the resolution and the associated uncertainty, the result is acceptable. We observe shifts of peak centers towards the desired energies with an increasing number of iterations. The LM algorithm is highlighted for its superior performance. The calibrated peaks in the energy spectrum are plotted in Figure 20. The Gaussian fits and the centers of the corresponding peaks are shown in Figure 21 and in Table 3, where we compare the results for several initial guesses.

## 5. Variable Projection

Inspired by the work on variable projection [24], we introduce a method, which moves the calibration partially towards analytical solution. Suppose that the optimal cost function depends only on $\beta_{d}$. Then, $\beta_{a}$, $\beta_{c}$, and $\beta_{d}$ are given as the solution to the linear least squares problem with dependence on $\beta_{d}$.

We define a reduced vector $\beta_{reduced}=\left(\beta_{a}\left(\beta_{d}\right),\beta_{b}\left(\beta_{d}\right),\beta_{c}\left(\beta_{d}\right)\right)$ satisfying the minimum of the cost function (2).

According to the derivations in Section 3, we can express the solution as follows:

(30) $$\left(\begin{array}{c} \sum y_{i}x_{i}\\ \sum y_{i}\\ \sum k_{i}y_{i} \end{array}\right)=\left(\begin{array}{ccc} \sum x_{i}^{2} & \sum x_{i} & -\sum x_{i}k_{i}\\ \sum x_{i} & n & -\sum k_{i}\\ \sum x_{i}k_{i} & \sum k_{i} & -\sum k_{i}^{2} \end{array}\right)\left(\begin{array}{c} \beta_{a}\\ \beta_{b}\\ \beta_{c} \end{array}\right)$$

where $k_{i}=\frac{1}{x_{i}-\beta_{d0}}$.

The associated reduced cost function is then written as follows:

(31) $$s_{nl}\left(\beta_{d}\right)=\sum_{i=1}^{n}{\left(y_{i}-\beta_{a}\left(\beta_{d}\right)x_{i}-\beta_{b}\left(\beta_{d}\right)+\frac{\beta_{c}\left(\beta_{d}\right)}{x_{i}-\beta_{d}}\right)}^{2}.$$

For $\forall \beta_{d}\in \left[0,x_{min}\right)$ the reduced cost function $s_{nl}$ is a differentiable function of the variable $\beta_{d}$.

If no stationary point lies in the interval $\left(0,x_{min}\right)$, the extrema of $s_{nl}\left(\beta_{d}\right)$ within the interval $\left[0,x_{min}\right)$ must lie on the boundary $\beta_{d}=0$ or $\beta_{d}\to x_{min}$. This is because $s_{nl}\left(\beta_{d}\right)$ is differentiable on the $\left[0,x_{min}\right)$ interval.

The variable projection method can be extended for $\beta_{d}$ < 0 by adding the additional condition ensuring E≥0 to the linear system with the variable $\beta_{reduced}$. The case $\beta_{c}=0$ corresponds to a linear fit.

**Figure 22:**
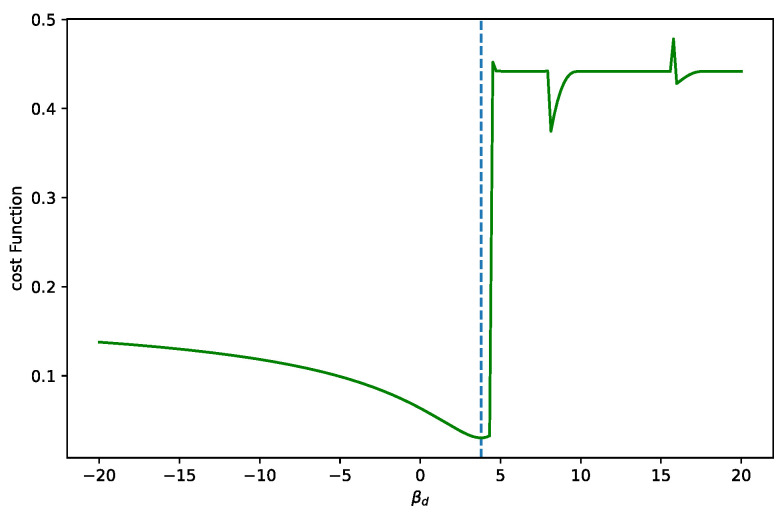
The cost function of the analytic solutions for varying $\beta_{d}$ values, while a good dataset is processed. Only one minimum is observed. In cases of poor data quality, we must ensure that $\beta_{d}>0$. The upper bound is given by the first calibration point. If $\beta_{d}>0$ exceeds this limit, the fit results in an infinite discontinuity. The dotted line marks to the minimum value.

We show the plots obtained by two types of datasets. The first dataset provides a minimum within the expected bounds. The cost function is shown in Figure 22. We can see that the calibration fit corresponds to the measured points.

**Figure 23:**
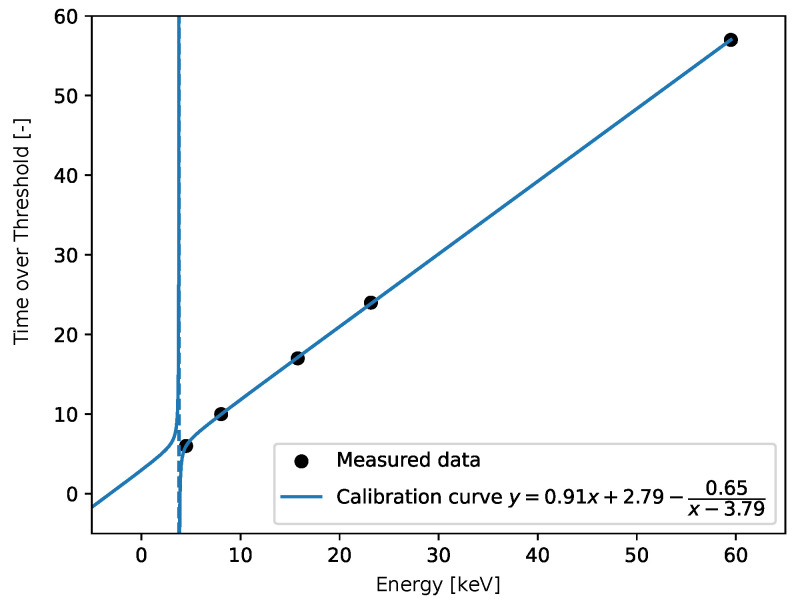
The fit for good data, the resulting equation is provided. The fit corresponds to the pixel (0, 3) in the related matrix plots. The blue dotted line indicates the discontinuity in the fit. Its position is given by the value of $\beta_{d}$.

**Figure 24:**
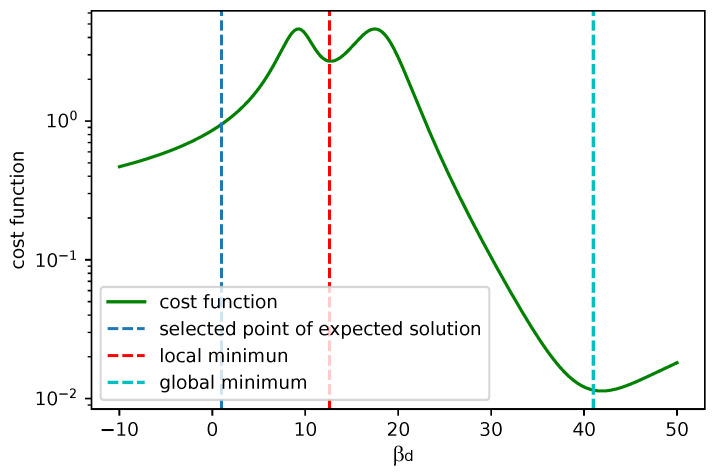
The cost function calculated analytically for given $\beta_{d}$ values. This example shows a case, where minima do not correspond to the proper fits. A suitable $\beta_{d}$ is around the blue line (corresponding picture is in Figure 25).

**Figure 25:**
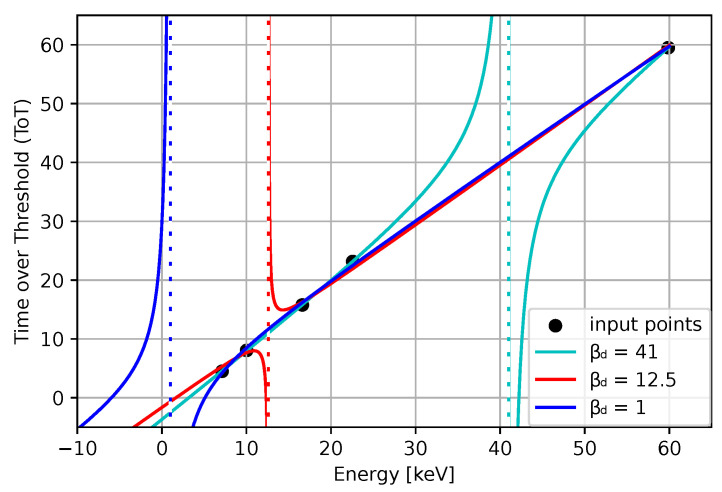
Fitted curves for fixed $\beta_{d}$. The red color ($\beta_{d}=12.5$) corresponds to a local minimum of the cost function, and the cyan curve ($\beta_{d}=41$) corresponds to the global minimum of the cost function. Both are incorrect, as is evident from the figure. Here we have to fix $\beta_{d}$ to the expected value, as shown in the blue function.

**Figure 26:**
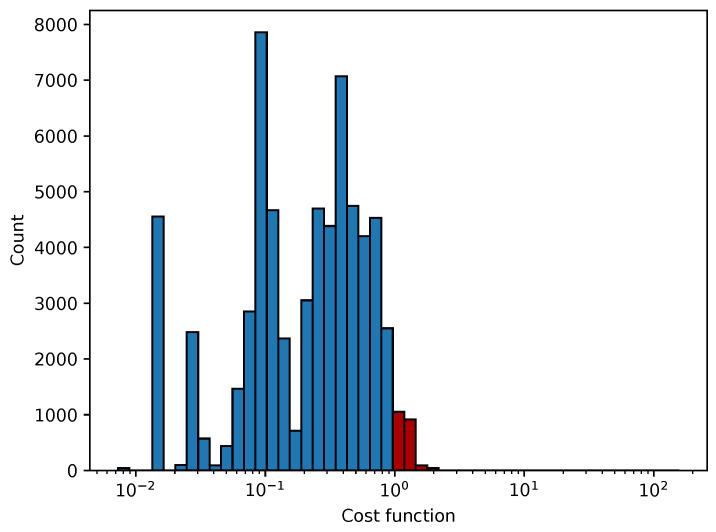
The cost function for all pixels when the variable projection method is used. The value $\beta_{c}$ is bounded to be larger than 0.

**Figure 27:**
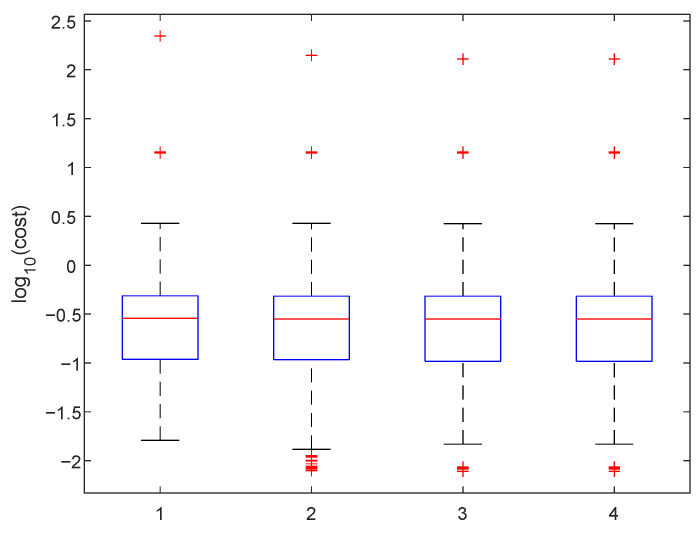
The box plot for the cost function. We show values respectively as follows: 1—LM (damping 1, adjusting constant 2), 10 iterations; 2—LM, 50 iterations; 3—LM—1000 iterations; and 4—variable projection, $\beta_{c}>0$.

**Figure 28:**
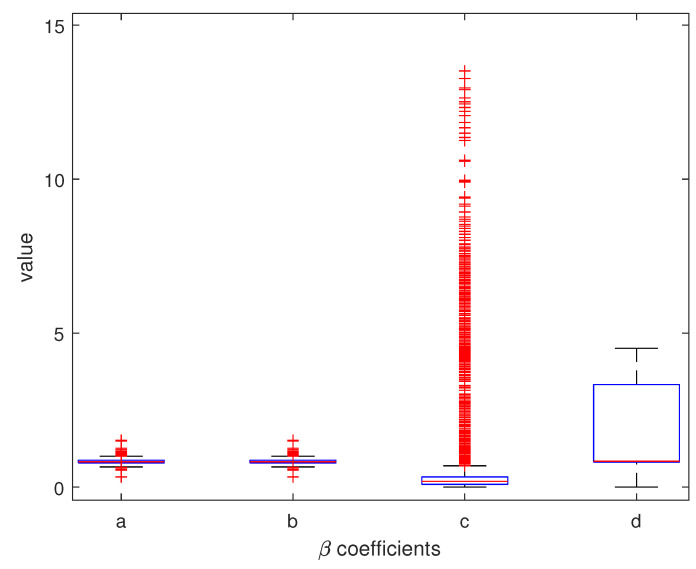
The box plot for $\beta_{a}$, $\beta_{b}$, $\beta_{c}$, and $\beta_{d}$ coefficients. The LM algorithm at 50 iterations and randomized initial guesses were used.

A good fit for 5 points is shown in Figure 23. For the practical use of the algorithm, we must ensure that $\beta_{c}$ is positive. In Figure 24 we can see two minima, local and global; however, both are out of bounds. The corresponding plot of the fits is shown in Figure 25. The red and cyan curves show the fits, which are not correct. If no minimum is found inside the given bounds, we must select a $\beta_{d}$ point within the bounds, reasonably close to the boundary with the minimal cost value. Then, we have to choose $\beta_{d}$ within the expected bounds. The plot of all pixels after the variable projection method is performed is shown in Figure 26. Statistical parameters for the LM algorithm and the variable projection method as a box plot are shown in Figure 27. The coefficient values are shown in Figure 28.

**Figure 29:**
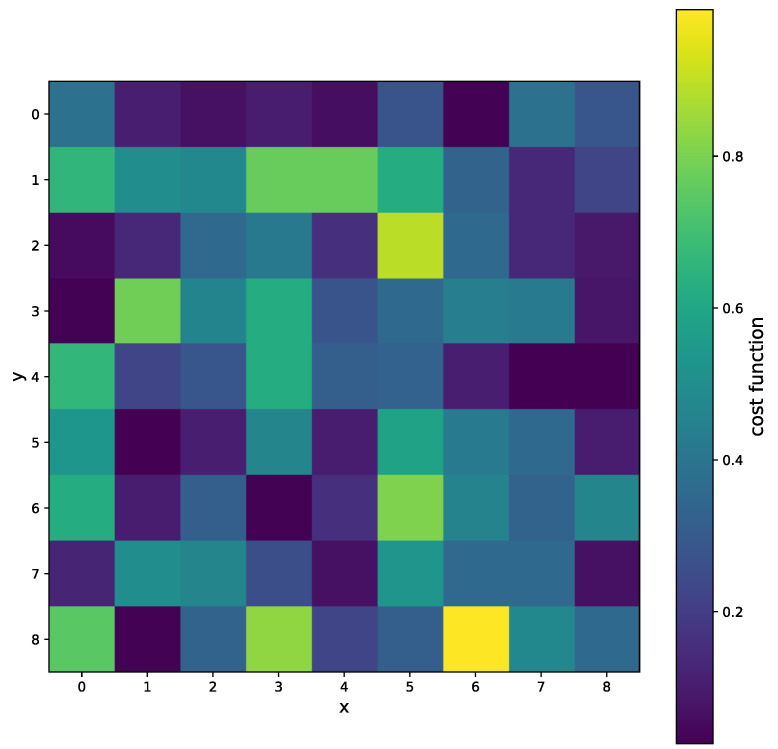
The selected area of the matrix, where *x* and *y* are pixel coordinates. The cost function for several pixels when the variable projection method is used.

**Figure 30:**
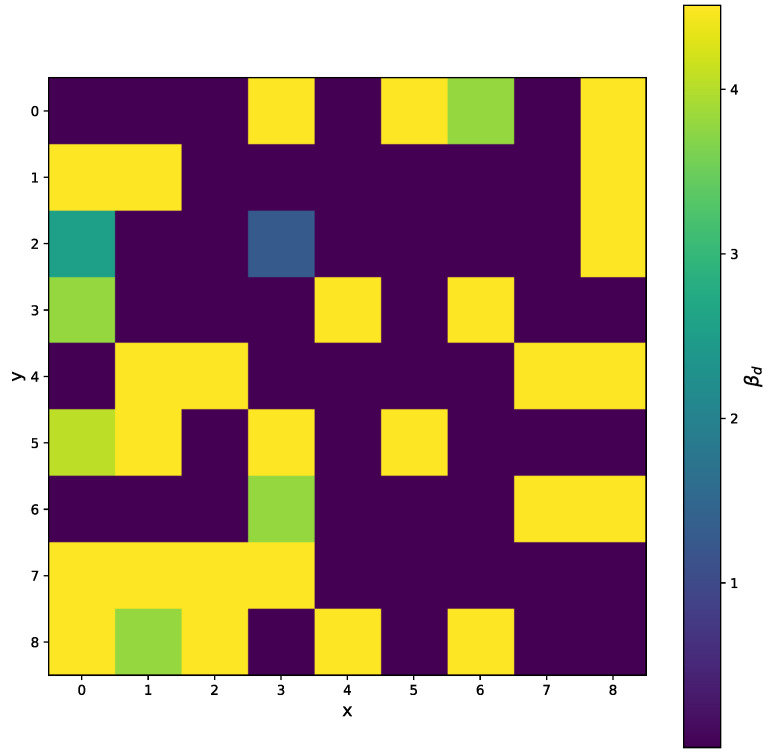
The selected area of the matrix, where *x* and *y* are pixel coordinates. The $\beta_{d}$ for all pixels when the variable projection method is used. We can see the range of $\beta_{d}$ values lower than the minimal measured energy point, corresponding to the bound (3).

**Figure 31:**
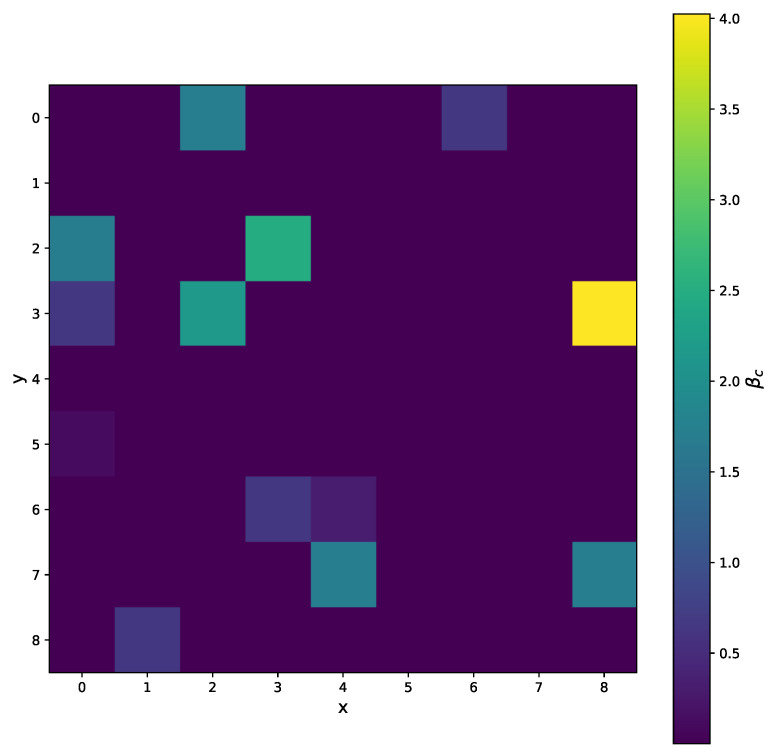
The selected area of the matrix, where *x* and *y* are pixel coordinates. The $\beta_{c}>0$ for all pixels when the variable projection method is used.

**Figure 32:**
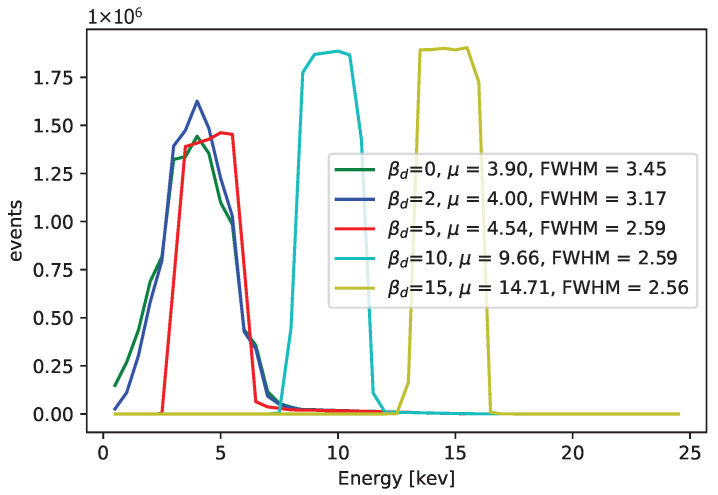
The comparison in terms of energy spectra for different $\beta_{d}$ values, when we fix this parameter (no other constrains were applied). Ti X-rays were used. The parameter $\beta_{d}$ was fixed to be the same constant for pixels. Better results can be obtained when the value $\beta_{d}$ is not fixed for all pixels and is instead determined by searching through the cost function. The cost function takes values around $1\times 10^{-1}$ for $\beta_{d}=2$ and $\beta_{d}=5$ and increases several times for $\beta_{d}=15$. The calibration points, different for each pixel, correspond to the peaks in the ToT values of each pixel.

The results for the variable projection method in the selected area of the matrix are shown in Figure 29, Figure 30 and Figure 31. The cost function and $\beta_{d}$ and $\beta_{c}$ values are plotted. The bound conditions are applied within the algorithm. The energy spectra for different $\beta_{d}$ values are depicted in Figure 32, where Ti is used as the X-ray source. The plot in Figure 16 corresponds to the pixel coordinates (x,y)=(0,3).

## 6. Conclusions

In this article, we studied a calibration model commonly used for the Timepix detectors with several approaches to solve non-linear regression problems. We show the convergence of the algorithms in terms of the cost function dependence on the iteration number and verify the area of initial guesses that converge to a good solution. Compared with the other two iterative algorithms, the LM regression with the proposed randomization tends to perform the best in all tested cases. The convergence trajectories for the LM algorithm were also plotted. The modified version with the additional randomization provided better results. Generally, the LM algorithm applied together with bounds checking is highly recommended to use. The GD algorithm is an abstract algorithm, not limited to solving least squares problems. Its convergence is slower and more dependent on the parameters compared to the LM algorithm.

As an alternative approach, we have also shown that the calibration can be solved as a variable projection into a one-dimensional optimization problem. For the given $\beta_{d}$ values we can calculate $\beta_{a}$, $\beta_{b}$, and $\beta_{c}$ as the least squares problem solution. The $\beta_{d}$ value must be lower than the first measured energy. The main advantage of this method is that it does not need the initial guesses as the algorithm input, while the iterative algorithms need guesses in all their modifications. Moreover, it can resolve convergence problems of the iterative algorithms.

The future work will be focused on the speed optimizations and implementation of the accelerated version using parallel algorithms. Moreover, $\chi^{2}$ minimization is a method worth considering for studies and comparisons.

## Figures and Tables

**Table 1:** Selected characteristic X-rays commonly used for calibration.

Material	$K\alpha_{1}$
Ti	4.51 keV
Cu	8.05 keV
Zr	15.78 keV
Cd	23.17 keV
In	24.21 keV

**Table 2:** Calibration sources.

Material	γ Ray Energy
Am-241	59.6, 26.3, 20.7 keV
Fe-55	5.89 keV

**Table 3:** Selected peak centers after applying the LM algorithm with 5 iterations. The last column corresponds to 50 iterations of the LM algorithm. The damping parameter was set to 1.

$\beta_{0}:$	(0,0,20,1)	(0,0,50,1)	(0,0,50,5)	(0,0,0,0)	(0,0,50,5), 50 it.
Ti	4.23 keV	4.5 keV	8.15 keV	3.29 keV	4.96 keV
Cu	7.55 keV	7.64 keV	9.82 keV	7.58 keV	7.54 keV
Zr	15.10 keV	14.78 keV	16.17 keV	15.83 keV	15.59 keV
Cd	22.26 keV	21.8 keV	22.99 keV	23.06 keV	22.90 keV
In	59.27 keV	59.52 keV	58.49 keV	58.84 keV	58.97 keV

## Data Availability

The data are currently not publicly available. Please contact the authors for access.

## Abbreviations

The following abbreviations are used in this manuscript:

ToT	Time-over-Threshold
GD	Gradient Descent
LM	Levenberg–Marquardt
GN	Gauss–Newton
RSS	Residual Sum of Squares
FWHM	Full Width at Half Maximum
